# Effects of Red Seaweed, Psyllium Husk, and Chia Seeds on Structural and Functional Properties of Meat Batters

**DOI:** 10.3390/foods15132385

**Published:** 2026-07-04

**Authors:** Milena Conte, Benjamin M. Bohrer

**Affiliations:** Department of Animal Sciences, The Ohio State University, Columbus, OH 43210, USA; conte.95@osu.edu

**Keywords:** comminuted meat, dietary fiber, meat ingredients, meat structure, physiochemical properties

## Abstract

The effects of red seaweed, psyllium husk, and chia seeds on the structural and functional properties of comminuted meat batters were evaluated. Meat batters were formulated with 1% of each ingredient or their combinations totaling 1% and evaluated for pH, cooking loss, microstructure, texture profile analysis, color, rheology, and protein interactions. Formulation did not affect (*p* ≥ 0.08) pH or cooking loss, indicating that water- and lipid-holding capacity and emulsion stability were preserved across treatments. Hardness increased (*p* ≤ 0.05) in treatments containing red seaweed, alone or combined with psyllium husk. Fiber addition did not influence (*p* ≥ 0.17) raw batter color; however, cooked products showed differences (*p* ≤ 0.05) in lightness (L*) and total color change (ΔE*). Rheological analysis indicated similar viscoelastic behavior among treatments with no significant differences among treatments (*p* ≥ 0.07) for storage modulus, loss modulus, or tangent delta at the start, peak, or end of the small-amplitude oscillatory shear test. Microstructural observations revealed treatment-dependent networks, and protein solubility analysis showed changes (*p* ≤ 0.05) in ionic and hydrogen bonding, while disulfide bonds were unaffected (*p* = 0.60). Incorporation of 1% of these ingredients maintained desirable physicochemical, textural, and functional properties, highlighting their potential as ingredients in meat batters.

## 1. Introduction

Fiber-rich diets have been shown to reduce the risk of health conditions such as type 2 diabetes, obesity, hypertension, and digestive disorders [1]. The recommended daily intake of dietary fiber ranges from 21 to 38 g per day (depending on sex and age) or approximately 14 g per 1000 kcal consumed. However, these intake levels are rarely achieved, particularly among individuals consuming Western diets [2]; thus, an opportunity exists to formulate commonly consumed food products with ingredients high in dietary fiber content.

Dietary fiber consists of complex plant-derived polysaccharides, including cellulose, non-cellulosic polysaccharides (e.g., mucilage, pectins, and hemicelluloses), and non-carbohydrate components such as lignin and gums [3,4]. Dietary fiber is commonly classified based on water solubility, viscosity, and fermentability into soluble dietary fiber and insoluble dietary fiber [5]. Soluble dietary fiber primarily includes pectins, gums, and mucilages [6]. Mucilages are seed-secreted mucopolysaccharides found in seeds such as chia, basil, and psyllium, and are characterized by their ability to form a visible gel-like coating upon hydration [7]. Red seaweed, psyllium, and chia seeds are technologically relevant sources of functional dietary fiber with significant potential in processed meat systems due to their water-holding, gelling, and textural properties. Red seaweed species such as *Gigartina skottsbergii* contain carrageenan, a group of sulfated galactans composed of alternating β-D- and α-D-galactose units. These compounds form thermoreversible gels whose strength and stability are strongly influenced by ionic conditions, particularly the presence of Na^+^, K^+^, and Ca^2+^. As a result, carrageenan can effectively bind water, stabilize protein–lipid emulsions, and improve texture and sliceability in comminuted and restructured meat products while also being associated with antioxidant and metabolic health benefits [8,9]. Psyllium husk consists primarily of highly branched arabinoxylans with β(1 → 4) and β(1 → 3) glycosidic linkages and a high degree of polymerization, enabling rapid hydration and the formation of viscous gels that enhance moisture retention and matrix integrity in food systems [7,10]. Similarly, chia seed mucilage is a high-molecular-weight glucoxylan rich in D-xylose, D-glucose, and glucuronic acid residues that forms a viscous coating upon hydration, contributing to emulsifying capacity and water retention. Collectively, these ingredients provide multifunctional properties that can improve yield, texture, and stability in processed meat formulations while also increasing dietary fiber content [11,12].

Beyond their health benefits, dietary fiber plays an important technological role in processed meat products by influencing key quality attributes such as water-holding capacity, viscosity, gel formation, and binding capacity [13]. Model systems, such as comminuted meat batters, are valuable tools for understanding how protein structure and functionality are affected by the incorporation of novel ingredients and how these interactions evolve during processing [14]. Among processing steps, thermal treatment is the most critical for promoting gel formation in comminuted meat systems [15]. From a rheological perspective, gel development is strongly influenced by the molecular characteristics of incorporated ingredients, including molecular weight, degree of branching, and structural complexity [7,16].

Despite extensive evidence supporting the nutritional and functional benefits of dietary fiber, limited information is available regarding how natural sources such as red seaweed, psyllium husk, and chia seed mucilage influence the structural and functional properties of comminuted meat systems. Therefore, the objective of this study was to evaluate the effects of red seaweed, psyllium husk, and chia seed on the structural and functional properties of comminuted meat batters.

## 2. Materials and Methods

### 2.1. Treatment Description

The three dietary fiber ingredients evaluated in this study were red seaweed (Nutricargo, Clifton, NJ, USA), psyllium husk powder (Kate Naturals, Irvine, CA, USA), and ground chia seeds (Worldwide Botanicals, Santa Barbara, CA, USA). According to manufacturer-provided nutritional information, red seaweed contained 10 g/100 g dietary fiber, 5 g/100 g protein, and 20 g/100 g carbohydrates; psyllium husk powder contained 80 g/100 g dietary fiber, 0 g/100 g protein, and 80 g/100 g carbohydrates; and ground chia seeds contained 42 g/100 g dietary fiber, 25 g/100 g protein, and 41.7 g/100 g carbohydrates. In the United States, nutrition labeling compliance is regulated by the U.S. Food and Drug Administration under 21 CFR §101.9 (g) [17]. For nutrients such as dietary fiber and protein, analytically determined values must generally be at least 80% of the declared label value, whereas nutrients that may not exceed declared values, such as calories, total fat, saturated fat, cholesterol, and sodium, must remain within established regulatory tolerances. Because the nutritional information reported for the dietary fiber ingredients was obtained directly from product labels and manufacturer specifications, these values were assumed to be compliant with applicable labeling regulations in the United States.

The pH values of red seaweed, psyllium husk powder, and ground chia seeds were 5.99 ± 0.06, 5.95 ± 0.06, and 5.94 ± 0.05, respectively. Lean beef (inside rounds–semimembranosus and associated muscles; 3.93% lipid) was portioned into 2.5 cm cubes, mixed, and ground using an 8 mm plate (Hobart 4732A; Hobart Corp., Troy, OH, USA). All visible connective tissue and fat were removed prior to grinding. Pork subcutaneous fat was similarly cut into 2.5 cm cubes and ground using the same plate. Following grinding, lean beef and pork fat were kept separate, vacuum-packaged in 400 g and 50 g portions, respectively, and stored at −20 °C until use. Non-meat ingredients included sodium chloride (Top-Flo Granulated; Cargill Ingredient Solutions, Cargill Inc. Minneapolis, MN, USA), sodium tripolyphosphate (Budenheim KG, Mansfield, OH, USA), Prague powder #1 [6.25% sodium nitrite and 93.75% NaCl; Newlyweds Foods, Chicago, IL, USA], and sodium erythorbate (Miller Scales & Food Machines, Fort Wayne, IN, USA).

Eight treatment formulations were evaluated: a control with no dietary fiber (CON); 1% red seaweed (SEA), 1% psyllium husk (PSY), or 1% chia seed (CHI); 0.5% red seaweed + 0.5% psyllium husk (SEA/PSY), 0.5% red seaweed + 0.5% chia seed (SEA/CHI), or 0.5% psyllium husk + 0.5% chia seed (PSY/CHI); and 0.33% red seaweed + 0.33% psyllium husk + 0.33% chia seed (SEA/PSY/CHI). All formulations are presented in Table 1. Treatments were independently prepared across three days (eight treatments per day) over three consecutive weeks, resulting in three independent replications per treatment (24 total experimental units).

### 2.2. Batter Preparation

Batter preparation followed the method of Bohrer et al. [18], with a few minor modifications. Each batch (500 g) was prepared using a food blender (Ninja BL770; SharkNinja Operating LLC, Needham, MA, USA) in a refrigerated room. Lean beef was chopped at the low-speed setting for 10 s, followed by the addition of sodium chloride, sodium tripolyphosphate, sodium nitrite, and half of the water (as ice), and chopped at the high-speed setting for 30 s. An additional 90 s of chopping was applied for protein extraction. Pork fat was then added and chopped for 90 s at the high-speed setting. Sodium erythorbate was added and mixed for 5 s. The remaining water was then incorporated, followed by 60 s of chopping at the high-speed setting. For the CON samples, water was added directly in liquid form, whereas for the fiber treatments, the dietary fiber ingredients were pre-suspended in half of the formulation water and manually stirred for 2 min until complete hydration was achieved. Batter temperature was monitored using a probe thermometer (Taylor USA, Oak Brook, IL, USA) to ensure it did not exceed 10 °C during the blending process. Batters were transferred into 50 mL polypropylene conical tubes (30 × 115 mm) using disposable piping bags and centrifuged at 1500× *g* for 5 min to remove air bubbles.

### 2.3. pH, Cooking Loss and Proximate Composition

The pH of raw batters was measured in triplicate using a benchtop pH meter (Hanna FC2323, Hanna Instruments, Smithfield, RI, USA), with the electrode fully immersed in the sample. After centrifugation, tubes were placed in a preheated water bath (Thermo Fisher Scientific, Waltham, MA, USA) at 80 °C to determine cooking loss. Internal temperature was monitored using a probe inserted into a non-experimental sample. Cooking was completed when samples reached 72 °C, after which tubes were cooled in ice water. Tubes were then inverted after lid removal. Proximate composition of cooked samples was determined using AOAC (2016) [19] methods for moisture (950.46), fat (991.36), protein (992.15), and ash (920.153). Moisture was determined by drying at 105 °C for 24 h in a forced-air oven. Lipid content was measured via Soxhlet extraction using chloroform:methanol (87:13). Protein content was determined using a combustion analyzer (Rapid N exceed; Elementar, Ronkonkoma, NY, USA). Ash content was measured using a muffle furnace (Thermolyne, Thermo Fisher Scientific Inc., Dubuque, IA, USA).

### 2.4. Microstructure

Microstructure was evaluated following procedures outlined in Bohrer et al. [18]. Samples were fixed in 10% neutral buffered formalin, dehydrated in graded ethanol solutions (70%, 95%, and 100%), embedded in paraffin, sectioned (4–6 μm), and stained with hematoxylin–eosin and periodic acid–Schiff reagents. Samples were observed at 4× magnification using a light microscope equipped with a high-resolution camera (Moticam X3, Kowloon, Hong Kong), and images were captured using imaging software (MotiConnect App version 1.5.2, Kowloon, Hong Kong).

### 2.5. Texture Profile Analysis

Cooked samples were evaluated for hardness (N), chewiness, cohesiveness (%), adhesiveness (g·s), and resilience (%). Samples were prepared as five cores (10 mm height × 30 mm diameter). Analysis was conducted using a texture analyzer (TA.XT plus100C; Stable Micro Systems, Hamilton, MA, USA) equipped with a 51 mm cylindrical probe (TA-25A). Pre-test and test speeds were set at 5.00 mm/s. The interval between the first and second compression cycles was 5 s, during which the probe returned to its initial position above the sample. The five cores for each sample were compressed twice to 50% of their original height with a trigger force of 50 g. For computational purposes, average values from the five cores were used.

### 2.6. Evaluation of Instrumental Color

Color of raw and cooked batters was measured using a spectrophotometer (Konica Minolta CM-700d, Osaka, Japan) with a D_65_ illuminant and 0° observer angle. Results were expressed as L*, a*, and b* values. Three measurements were recorded per sample. Both surface and internal color were evaluated for cooked samples. Total color change (ΔE*) relative to the CON samples was calculated using Equation (1).

(1) $$Totalcolorchange\left(\Delta E*\right)=\sqrt{(SampleL^{*}-BaselineL^{*}{)}^{2}+(Samplea^{*}-Baselinea^{*}{)}^{2}+(Sampleb^{*}-Baselineb^{*}{)}^{2}}$$

### 2.7. Dynamic Rheology

Small-amplitude oscillatory shear testing was conducted using a rheometer (Discovery HR-3; TA Instruments, New Castle, Delaware, USA) with parallel-plate geometry (40 mm diameter, 2 mm gap). A 50 mm loading gap and a frequency of 1 Hz were used. Sample edges were coated with silicone oil (~60,000 mPa·s) to prevent drying. Samples were equilibrated at 20 °C for 3 min, heated to 72 °C at 1.5 °C/min, and then cooled to 20 °C at the same rate (total run time ~72 min). Storage modulus (G′), loss modulus (G″), and tangent delta (tan δ) were continuously recorded throughout the thermal ramp in triplicate. For statistical analysis, rheological parameters were extracted at three predefined temperature points representing the start of heating (20 °C), peak heating (72 °C), and the end of cooling (20 °C). Only these extracted values were subjected to statistical analysis and treatment comparisons; the complete rheological curves were used for graphical visualization and qualitative interpretation of gelation behavior.

### 2.8. Molecular Forces

Molecular forces were evaluated following Yuan et al. [20] and Wang et al. [21]. Ionic, hydrogen, hydrophobic, and disulfide interactions were assessed using NaSCN (0.5 M), urea (8 M), SDS (0.5% *w*/*v*), and β-mercaptoethanol (0.25% *v*/*v*), respectively. Samples (~0.11 g) were homogenized with 1 mL of each solution using a bead mill homogenizer (Thermo Fisher Scientific) for 200 s. Samples were heated (80 °C, 1 h), cooled to room temperature, and centrifuged (10,000× *g*, 15 min). Supernatants were collected and stored at −80 °C. Protein solubility was calculated based on protein concentration in the supernatant relative to the original homogenate. Protein concentration was determined using the Compat-Able™ reagent set (Thermo Fisher Scientific Inc., Dubuque, IA, USA) and Pierce™ BCA assay (Thermo Fisher Scientific Inc., Dubuque, IA, USA).

### 2.9. Statistical Analysis

Proximate composition was summarized using arithmetic means and standard deviation. All other data were analyzed as a randomized complete block design using PROC GLIMMIX in SAS (SAS 9.4; SAS Institute Inc., Cary, NC, USA). Treatment served as the fixed effect, and replication served as a random effect. When appropriate, the residual covariance structure was assumed to be independent (variance components), as measurements were analyzed as single observations per experimental unit rather than repeated measures. Least squares means were generated using the LSMEANS statement, and pairwise comparisons were performed using the PDIFF option.

For rheological measurements, although storage modulus (G′), loss modulus (G″), and tan δ were continuously recorded across the thermal ramp, statistical analyses were conducted only on values extracted at predefined temperature points (20 °C initial, 72 °C peak heating, and 20 °C post-cooling). Therefore, no repeated-measures model or covariance structure across time was applied to the full rheological curves.

Model assumptions, including normality of residuals and homogeneity of variance, were evaluated using studentized residual plots and Shapiro–Wilk tests within PROC GLIMMIX. When necessary, data were examined for influential observations; however, no transformations were required as model assumptions were satisfactorily met for all response variables.

## 3. Results and Discussion

### 3.1. Determination of pH, Cooking Loss and Proximate Composition

The proximate composition of meat batters formulated with dietary fiber ingredients is presented in Table 2. Inclusion of dietary fiber ingredients and their combinations at the 1% inclusion level had minimal impact on proximate composition. Moisture content remained consistent among treatments, ranging from 70.34% (±0.24) to 71.03% (±0.08). Protein content ranged from 15.30% (±1.30) to 16.67% (±0.26), while lipid content varied from 6.31% (±1.43) to 7.88% (±1.50). Ash content remained stable at 2.86% (±0.24) to 3.41% (±0.07) across all formulations. These results indicate that dietary fiber inclusion maintained the overall composition of the meat batters.

It should be noted that the dietary fiber ingredients differed substantially in their fiber concentrations, with red seaweed, psyllium husk, and chia seed containing approximately 10%, 80%, and 42% dietary fiber, respectively. Consequently, a 1% ingredient inclusion level did not result in equivalent dietary fiber concentrations among treatments. The present study was designed to compare the functionality of these commercially available ingredients at equal formulation inclusion levels rather than at equivalent dietary fiber concentrations. Therefore, observed differences among treatments may reflect both differences in total dietary fiber contribution and differences in the physicochemical properties of the fiber sources themselves. Future research should evaluate these ingredients at standardized dietary fiber inclusion levels to better distinguish the effects of fiber concentration from ingredient-specific functionality.

The pH of raw meat batters ranged from 5.86 to 6.00 and was not affected by treatment (*p* = 0.43; Table 3), indicating that 1% inclusion of the dietary fiber ingredients did not influence batter pH. Previous research reported no pH changes in phosphate-free meat emulsions with up to 2.5% bamboo fiber, with effects only observed at 5% inclusion [22]. In contrast, reductions in pH of meat batters have been reported with carboxymethyl cellulose and methylcellulose due to their inherent acidity [18]. Huang et al. [23] demonstrated that ingredient interactions within comminuted meat systems may also influence pH, as native breadfruit flour increased batter pH despite having a lower inherent pH than extruded flour. These findings suggest that batter pH is influenced by inclusion level, ingredient properties, and interactions between ingredients and meat systems.

Cooking loss was not affected by treatment (*p* = 0.08), with all values below 0.70%, indicating strong emulsion stability and water- and lipid-holding capacity during thermal processing [24]. Although dietary fiber ingredients are often associated with improved water-holding capacity, reported effects on cooking loss vary depending on fiber type and formulation [23,25,26,27]. In this study, pH remained sufficiently distant from the isoelectric point of meat proteins, and the presence of phosphates [28] likely contributed to a stable protein matrix through enhanced water-holding capacity and emulsification, which resulted in consistently low levels of cooking loss.

### 3.2. Microstructure

**Figure 1:**
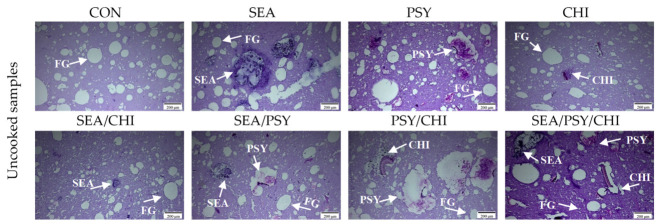
Light micrographs of raw (uncooked) meat batters formulated without dietary fiber (CON) or with 1% inclusion of red seaweed (SEA), psyllium husk (PSY), and/or ground chia seeds (CHI); Scale bar = 200 μm.

**Figure 2:**
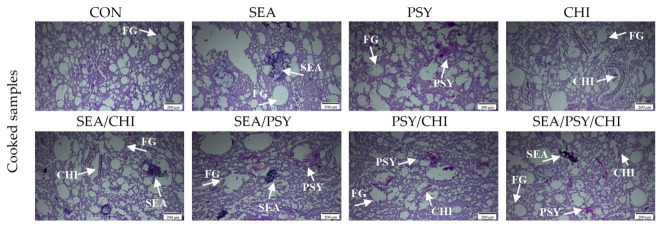
Light micrographs of cooked meat batters formulated without dietary fiber (CON) or with 1% inclusion of red seaweed (SEA), psyllium husk (PSY), and/or ground chia seeds (CHI); Scale bar = 200 μm.

The microstructural characteristics of the batters were observed using a light microscope, as shown in Figure 1 and Figure 2. The protein and lipid phases were dyed magenta to focus on the identification of polysaccharides; in this case, the dietary fiber ingredients and their derivatives, as well as their protein–lipid–water interactions. The lipid globules are represented by semi-round shapes with a white color. The magenta-dyed background represents the protein phase, and it was possible to observe dark magenta structures of varying shapes. For the CON samples, no dark magenta structures were presumably found because no dietary fiber ingredients were included in these formulations. For the SEA treatments, a multilobular dark magenta structure was presumed to be the red seaweed, and this structure was represented in both uncooked and cooked SEA, SEA/CHI, SEA/PSY, and SEA/PSY/CHI treatments. For the CHI treatments, the seed coat was presumed to be observed, and it is likely the mucilage has been released to surround the seed, in small pieces, possibly due to the physical processing involved with the comminuted samples. The seed coat structures were presumably observed in the CHI, SEA/CHI, PSY/CHI, and SEA/PSY/CHI treatments. Treatments containing psyllium (PSY, SEA/PSY, PSY/CHI, and SEA/PSY/CHI) were represented with light magenta color and were presumed to contain a veil-like structure, which was unique compared to the other dietary fiber treatments.

### 3.3. Texture Profile Analysis (TPA)

Instrumental texture measurements are closely associated with sensory perception [29]. The incorporation of dietary fiber ingredients significantly affected (*p* = 0.001) hardness in the present study. Treatments containing SEA, particularly SEA and SEA/PSY, exhibited the greatest hardness and differed (*p* ≤ 0.05) from CON, PSY, CHI, PSY/CHI, and SEA/PSY/CHI. This increase may be attributed to reinforcement of the protein gel matrix. Zhuang et al. [30] reported that insoluble dietary fiber (≥0.5%) enhances gel strength by promoting a “trapped” network structure. Similarly, Jiang et al. [31] observed denser gel networks and increased hardness in chicken meat gels with red algae inclusion. Seaweed-based ingredients have been shown to improve hardness, cohesiveness, and chewiness due to enhanced water- and lipid- holding capacity [32]. Yuan et al. [20] reported increased hardness in phosphate-free sausages with 1.25% red seaweed fiber. These findings support the role of seaweed in forming a more compact and stable protein network. In contrast, CHI, PSY/CHI, and SEA/PSY/CHI did not differ (*p* > 0.05) from the CON treatment for hardness, indicating that 1% inclusion did not alter gel strength in these treatments.

Interestingly, the increase in hardness was not accompanied by differences (*p* > 0.05) in cohesiveness or chewiness, which can be attributed to the distinct structural basis of these texture parameters. Hardness reflects the peak force required to deform the sample and is primarily influenced by the initial rigidity and resistance of the gel matrix, whereas cohesiveness represents the extent of internal structural integrity and the ability of the matrix to withstand a second deformation. The observed increase in hardness therefore likely reflects localized reinforcement of the protein network and physical filling effects, without substantially altering the fundamental protein–protein interactions responsible for structural cohesion and recovery. Likewise, chewiness is a composite parameter derived from hardness, cohesiveness, and springiness; thus, the absence of changes in cohesiveness and springiness limited the propagation of hardness differences into overall chewiness values. These results collectively suggest that dietary fiber modified the initial resistance of the meat batter matrix to compression rather than fundamentally altering internal network stability or elastic recovery behavior.

### 3.4. Color

Color is a key quality attribute influencing consumer acceptance of meat products. Inclusion of the dietary fiber ingredients did not affect (*p* ≥ 0.17) the color of raw batters (Table 4); however, several treatment effects (*p* ≤ 0.05) were observed in cooked samples. Only surface L* values differed (*p* = 0.05), with SEA/CHI exhibiting greater (*p* ≤ 0.05) lightness than PSY and CHI, while other treatments were at intermediate values. Total color change (ΔE*) of internal slices was also affected (*p* = 0.02), with SEA/CHI and SEA/PSY/CHI showing greater changes than CHI, SEA/PSY, and PSY/CHI.

Previous research indicates color effects depend on ingredient characteristics. Seaweed inclusion has been associated with increased lightness in frankfurters [20], which may be related not only to dilution effects from the added ingredient but also to the presence of natural pigments in seaweed, including chlorophyll derivatives, carotenoids, and, in red algae, phycobiliproteins such as phycoerythrin [33]. These pigments can influence light absorption and scattering behavior in meat matrices and may therefore contribute to observed differences in L* values and overall color stability during heating. Chia seed has been reported to have minimal impact on color at moderate inclusion levels [34]. In the present study, PSY treatments exhibited lower L* values (*p* < 0.05), indicating darker products, which are likely due to the natural color of the psyllium husk ingredient (b* 21.99 ± 0.42) [35]. Similar trends have been reported for psyllium and other plant-based ingredients [36,37,38].

Color change (ΔE*) values ranged from 1.83 to 2.94, indicating noticeable but acceptable differences, as values ≥3 are typically considered unacceptable differences in color change [39].

### 3.5. Rheology

**Figure 3:**
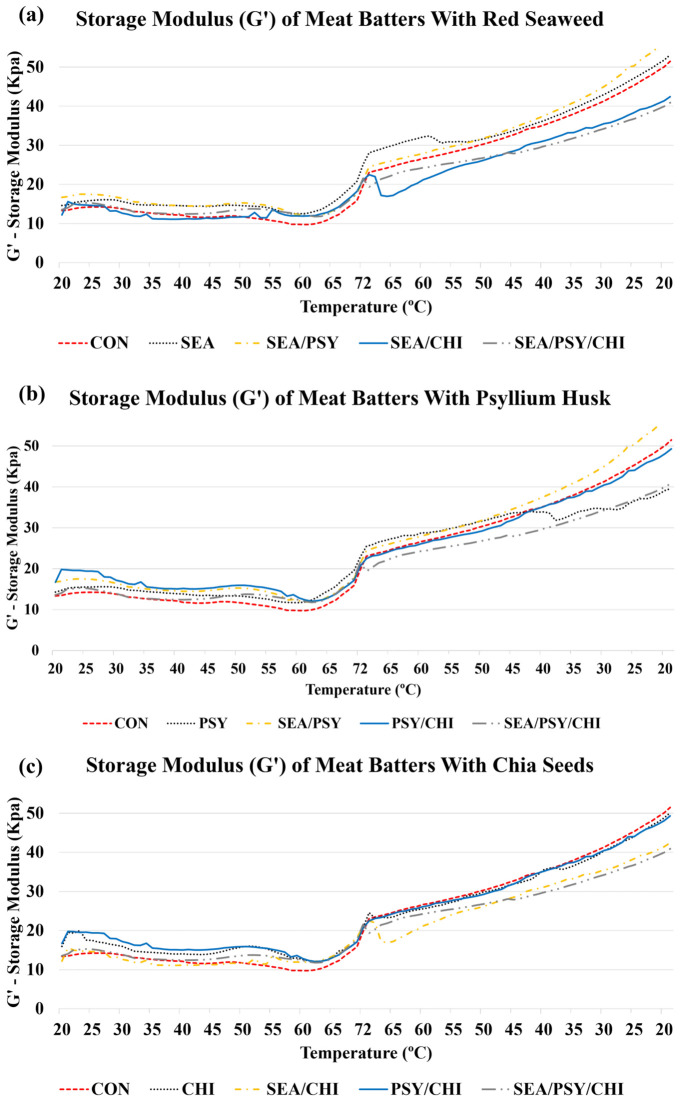
Storage modulus (G′) of meat batters formulated at a total of 1% of red seaweed (SEA), psyllium husk (PSY), and chia seeds (CHI), and their combinations and without dietary fiber addition (CON)—(**a**), SEA treatments and CON—(**b**), PSY treatments and CON—(**c**), CHI treatments and CON. Average of the triplicate samples started at 20 °C, heated to 72 °C, and then cooled to 20 °C.

**Figure 4:**
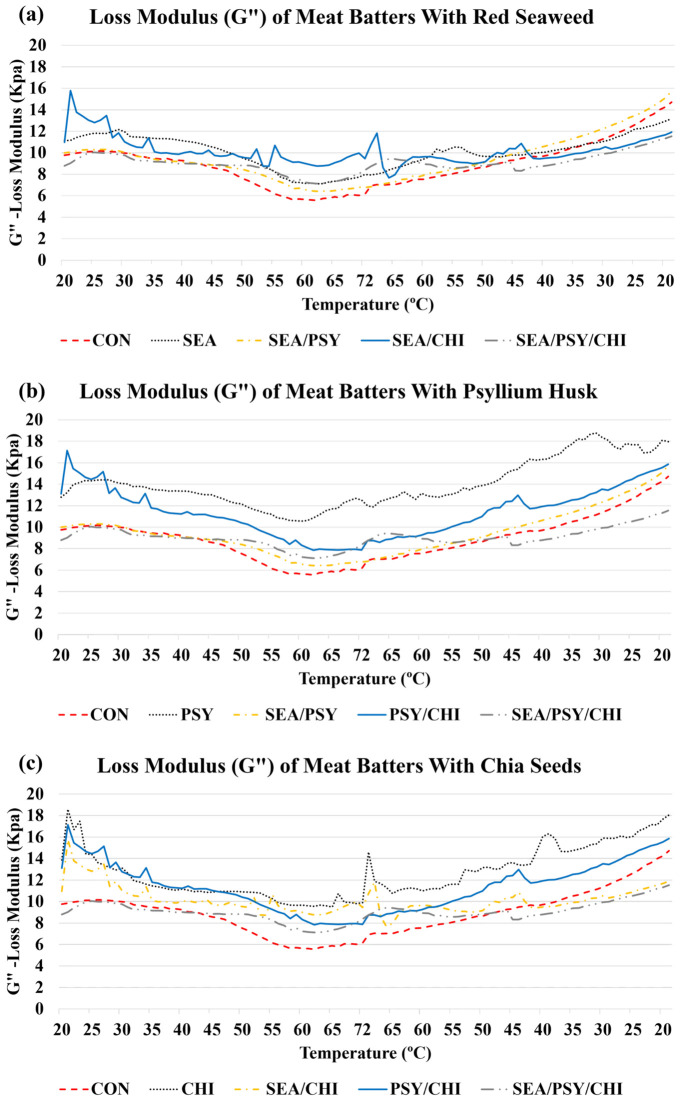
Loss modulus (G″) of meat batters formulated at a total of 1% of red seaweed (SEA), psyllium husk (PSY), chia seeds (CHI), and their combinations and without dietary fiber addition (CON)—(**a**), SEA treatments and CON—(**b**), PSY treatments and CON—(**c**), CHI treatments and CON. Average of the triplicate samples started at 20 °C, heated to 72 °C, and then cooled to 20 °C.

**Figure 5:**
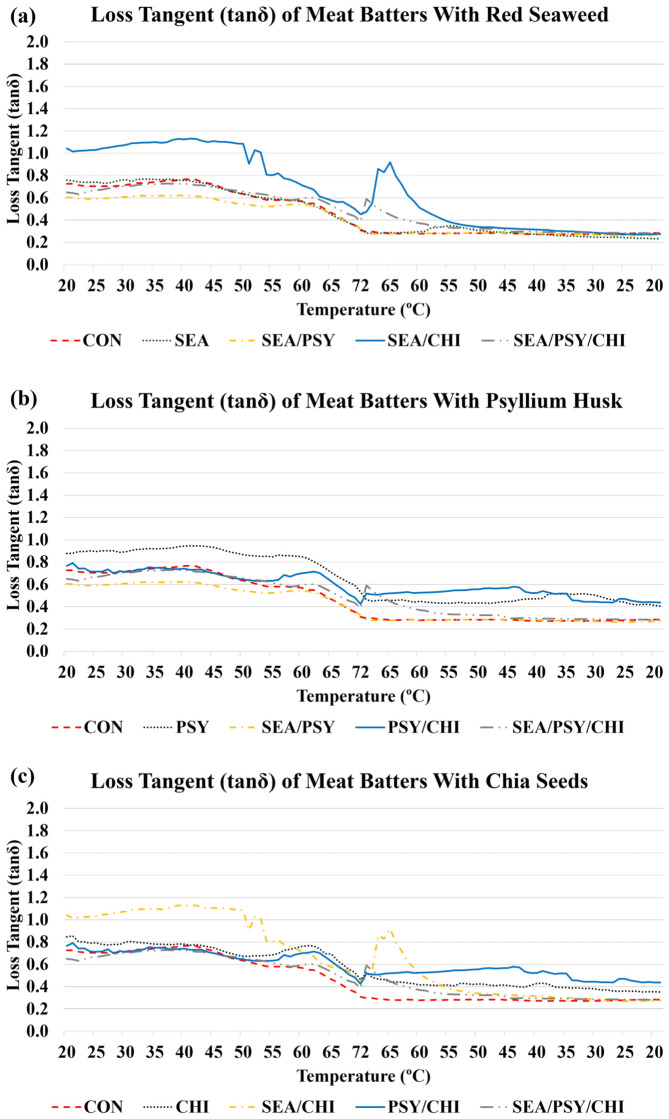
Tangent delta (tan δ) of meat batters formulated at a total of 1% of red seaweed (SEA), psyllium husk (PSY), chia seeds (CHI), and their combinations and without dietary fiber addition (CON)—(**a**), SEA treatments and CON—(**b**), PSY treatments and CON—(**c**), CHI treatments and CON. Average of the triplicate samples started at 20 °C, heated to 72 °C, and then cooled to 20 °C.

Dynamic rheological measurements provide insight into protein gelation and network formation, particularly in meat batters, where they provide insight into protein gelation and network formation during thermal processing. Treatments were assessed using two temperature ramps: an initial heating phase from 20 °C to 72 °C, followed by a cooling phase from 72 °C to 20 °C. The storage modulus (G′), loss modulus (G″), and tangent delta (δ) of the meat batters are presented in Table 5. The numerical rheological measurements support the trends observed in Figure 3, Figure 4 and Figure 5 (described below).

At the start of heating (20 °C), no differences (*p* > 0.05) were observed among treatments for G′, G″, or tan δ, indicating that dietary fiber inclusion did not alter the initial viscoelastic properties of the uncooked meat batters. During heating to 72 °C, G′ values increased numerically from 19.69 kPa in CON to 25.04 kPa in SEA, although differences were not significant (*p* = 0.28). Likewise, G″ values at 72 °C ranged from 6.13 to 12.52 kPa and did not differ among treatments (*p* = 0.46). Following cooling to 20 °C, all treatments exhibited substantially greater G′ values compared with the initial measurements, demonstrating the formation of a thermally induced protein gel network. Numerically, the SEA/PSY treatment exhibited the greatest post-cooling G′ value (58.21 kPa), whereas PSY (40.00 kPa) and SEA/PSY/CHI (41.14 kPa) exhibited the lowest values; however, these differences were not significant (*p* = 0.54). Although no statistical differences were observed for final storage modulus (G′) after cooling, numerical differences among treatments were evident. Relative to the control treatment, SEA/PSY exhibited the greatest numerical increase in final G′ (+12.1%), whereas PSY (−23.0%) and SEA/PSY/CHI (−20.7%) showed lower values. These trends suggest that specific fiber combinations may influence the extent of network reinforcement during gel maturation, even in the absence of statistically detectable differences.

Similarly, post-cooling G″ values remained statistically similar among treatments (*p* = 0.84). Although most rheological parameters were unaffected by treatment, tan δ values after cooling tended to differ (*p* = 0.07), with SEA exhibiting the lowest value (0.23) and PSY/CHI the highest value (0.44). These numerical differences indicate that seaweed-containing treatments developed a more elastic gel structure relative to treatments containing psyllium, which is consistent with the rheological profiles throughout the rheology test and supports the conclusion that seaweed promoted stronger elastic network formation during thermal processing.

As the heating ramp started (Figure 3a–c), the first noticeable change occurred for all treatments around 28 °C, where G′ showed a slight decrease, indicating initial structural modifications in the meat batters. A more pronounced reduction in G′ and G″ was observed between 60 °C and 65 °C, reflecting a weakening of the matrix structure. Heat-induced gelation and the resulting textural properties of meat batters are largely governed by myofibrillar proteins, which constitute approximately 55–60% of the total muscle protein content [40]. Rheological analysis of pure myofibrillar protein gels suggests that a dimerization process of myosin heads starts between 40 °C and 50 °C (slight increase in G′), followed by unfolding of the myosin tail, which demonstrates transient gel weakening (temporary decrease of G′) [30]. These oscillations are consistent with myosin denaturation, which precedes the formation of gel networks [41,42]. In the present study, structural transitions reflected the development and stabilization of the protein matrix.

At the exact same time-temperature point of 72 °C (peak heating), the SEA treatment showed the greatest numerical (*p* = 0.28) G′ values among all treatments, consisting of a strong gel (defined by greater G′ than G″; Figure 4a–c). The SEA treatment had lower numerical (*p* = 0.07) tan δ values at the last point of 20 °C (after cooling), which corresponded to a predominance of elastic behavior as opposed to viscous behavior [43]. Similar trends have been reported in previous research for κ-carrageenan extracted from red seaweed, where G′ predominates over G″ across thermal profiles, supporting the formation of stable and elastic gels [44].

Correspondingly, the tangent delta (tan δ) decreased during the initial heating stage, followed by a more substantial drop between 60 °C and 72 °C (Figure 5a–c), suggesting the development of a more elastic network. G′ values stabilized beyond 72 °C (peak heating to post-cooling) and began to increase sharply during the cooling phase, indicating that the structural transition and protein network reorganization had reached completion and formation of a meat protein gel network occurred [41,45].

The PSY treatment had the greatest tan δ at 72 °C (peak heating), indicating less elasticity; however, the values were not different (*p* > 0.05) from the CON samples at 20 °C (after cooling). The SEA treatment had lower (*p* ≤ 0.05) tan δ values at 20 °C (after cooling), which corresponded to the predominance of elastic behavior over viscous behavior, which is generally related to highly structured materials and better gel quality. In addition, tan δ values may vary if greater heating rates are applied due to differences in protein gelation [15,43].

All trends for G′ and G″ were stable beyond 65 °C, and the increase in the G′ values as the cooling ramp started was likely due to structural transitions and conformational completion. Phases one, two, and three observed in this section align with research by [46], who reported that the definition of thermally induced gel matrix formation is summarized first by the denaturation step, second by the aggregation step, and third by cross-linking. The SEA treatment presented the most favorable rheological properties for gel strength and elasticity. Further studies are needed to evaluate the long-term stability of these gel systems.

### 3.6. Molecular Forces

Molecular interactions in meat batters containing dietary fiber ingredients are influenced by protein–polysaccharide interaction mechanisms, which play a key role in the stabilization of protein-based emulsions. Therefore, understanding changes in interaction mechanisms is essential to elucidate protein network formation and emulsion stability in these systems [47]. These molecular-level interactions ultimately manifest at the macroscopic scale as differences in viscoelastic behavior (i.e., storage modulus, loss modulus, and tan δ), since gel strength and elasticity are governed by the type, density, and stability of protein cross-linking within the heat-induced network.

Protein solubility assessed using selective disruptive reagents showed that the inclusion of different dietary fiber ingredients significantly affected the molecular interactions used to stabilize meat batters (Table 6). Protein solubility in the presence of sodium thiocyanate (NaSCN), an indication of ionic interactions, differed among treatments (*p* = 0.003). Batters formulated with CHI and SEA/CHI exhibited greater (*p* ≤ 0.05) values for solubility compared with the CON samples, suggesting a greater contribution of ionic interactions in these formulations.

Hydrogen bonding, evaluated through solubility in 8 M urea, was also influenced by dietary fiber inclusion (*p* = 0.04), yet the treatments were not different (*p* > 0.05) when compared with CON. Treatments containing CHI, either alone or in combination with SEA or PSY, showed greater (*p* ≤ 0.05) protein solubility. Previous studies have reported that red algae-based systems can promote hydrogen bonding and hydrophobic interactions while carrageenan–protein systems may form electrostatic complexes depending on pH conditions [31,48]. Overall, protein–polysaccharide interactions are commonly formed by electrostatic attraction and are strongly influenced by the structural characteristics of the polysaccharides, including molecular weight as well as processing conditions such as temperature and pH [49].

Protein solubility in sodium dodecyl sulfate (SDS), associated with hydrophobic interactions, did not differ among treatments (*p* = 0.47). These results suggest that hydrophobic interactions were not influenced by the inclusion of the ingredients. Cao et al. [45] indicated that hydrophobic interactions did not play a significant role in gel network formation. Protein functionality may still be enhanced through protein–polysaccharide complex formation, primarily driven by electrostatic and hydrogen bonding interactions [49].

Similarly, protein solubility in β-mercaptoethanol (β-ME), an indication of disulfide bond disruption, was not affected by fiber addition (*p* = 0.60). This indicates that disulfide bond formation remained unchanged across treatments, suggesting that the incorporation of dietary fiber ingredients in this study did not interfere with covalent cross-linking mechanisms involved in protein gelation in which disulfide bonds play a major structural role [35]. Chickpea dietary fiber showed a different effect in the pork myofibrillar protein gels exposed to more sulfhydryl, and consequently, the surface hydrophobicity and gel density [50].

In a study conducted by Zhou et al. [37], psyllium husk at the 2% inclusion level was the ideal percentage for the gel network and its properties due to the promotion of disulfide bonds between the myofibrillar protein gels, while psyllium husk at the 3% inclusion level showed significantly lower disulfide bonds, which caused an overfilling effect that blocked the formation of the network and disrupted protein interactions. Protein interactions were not affected by the ingredients evaluated in this study. In the present study, psyllium-containing treatments did not alter chemical bonding across disruptive reagents, which, together with rheological results, suggests that the inclusion levels used were within a range that does not interfere with protein network formation but may contribute to subtle modifications in gel structure. Collectively, these molecular findings are consistent with the rheological responses observed, where differences in storage modulus (G′), particularly during heating and after cooling, reflect variations in the extent and stability of protein network formation driven primarily by ionic and hydrogen bonding interactions rather than changes in hydrophobic or disulfide bonding.

A limitation of the present study is that molecular interactions were inferred from differential protein solubility in selective disruptive reagents rather than measured directly. Although this approach is widely used to estimate the relative contributions of ionic, hydrogen, hydrophobic, and disulfide interactions, the reagents may not be entirely specific to a single interaction type and can disrupt multiple molecular forces simultaneously. Consequently, the observed changes in protein solubility should be interpreted as indicators of the predominant interactions contributing to protein network stabilization rather than direct measurements of individual chemical bonds. Future studies employing complementary techniques such as Fourier-transform infrared spectroscopy (FTIR), Raman spectroscopy, or other structural analyses would provide additional confirmation of the mechanisms governing protein–polysaccharide interactions in these systems.

## 4. Conclusions

This study provides a foundation for the development of dietary fiber-enriched processed meat products. The feasibility of incorporating dietary fiber ingredients (red seaweed, psyllium husk, and chia seeds) at a 1% inclusion level was demonstrated in processed meat systems without compromising structural or physicochemical properties. All treatments maintained comparable water-holding capacity, cooking loss, and thermal behavior. Texture and rheological properties were preserved or slightly improved, with red seaweed contributing to enhanced gel strength. Among the ingredients evaluated, psyllium husk has the greatest dietary fiber content and did not alter the molecular interactions within the meat matrix, highlighting its promise as a functional ingredient that could be used to increase dietary fiber content in processed meat products. Further studies should investigate greater inclusion levels of psyllium husk and their effects on product quality and consumer-relevant attributes such as sensory preferences, digestibility, and nutritional potential.

## Figures and Tables

**Table 1:** Formulations for meat batters formulated without dietary fiber (CON) or with 1% inclusion of red seaweed (SEA), psyllium husk (PSY), and/or ground chia seeds (CHI).

Ingredients	CON	SEA	PSY	CHI	SEA/PSY	SEA/CHI	PSY/CHI	SEA/PSY/CHI
Ground beef, %	67.50	66.50	66.50	66.50	66.50	66.50	66.50	66.5
Pork back fat, %	10.00	10.00	10.00	10.00	10.00	10.00	10.00	10.00
Water, %	20.00	20.00	20.00	20.00	20.00	20.00	20.00	20.00
Sodium chloride, %	1.50	1.50	1.50	1.50	1.50	1.50	1.50	1.50
Sodium tri-polyphosphate, %	0.35	0.35	0.35	0.35	0.35	0.35	0.35	0.35
Prague powder #1, % (with 6.25% sodium nitrite)	0.15	0.15	0.15	0.15	0.15	0.15	0.15	0.15
Sodium erythorbate, %	0.50	0.50	0.50	0.50	0.50	0.50	0.50	0.50
Red seaweed ^1^, %	0.00	1.00	0.00	0.00	0.50	0.50	0.00	0.33
Psyllium husk ^2^, %	0.00	0.00	1.00	0.00	0.50	0.00	0.50	0.33
Chia seed ^3^, %	0.00	0.00	0.00	1.00	0.00	0.50	0.50	0.33

^1^ The red seaweed ingredient contained 10 g dietary fiber/100 g. ^2^ The psyllium husk ingredient contained 80 g dietary fiber/100 g. ^3^ The chia seed ingredient contained 42 g dietary fiber/100 g.

**Table 2:** Proximate composition of meat batters formulated without dietary fiber (CON) or with 1% inclusion of red seaweed (SEA), psyllium husk (PSY), and/or ground chia seeds (CHI) ^1^.

	CON	SEA	PSY	CHI	SEA/PSY	SEA/CHI	PSY/CHI	SEA/PSY/CHI
Moisture, %	71.03 ± 0.08	70.51 ± 0.36	70.46 ± 0.20	70.48 ± 0.20	70.56 ± 0.19	70.77 ± 0.03	70.91 ± 0.24	70.34 ± 0.24
Protein, %	16.03 ± 0.91	16.67 ± 0.26	15.90 ± 1.16	15.79 ± 0.05	15.78 ± 0.01	16.44 ± 0.52	15.30 ± 1.30	15.82 ± 0.20
Lipid, %	7.75 ± 0.72	6.57 ± 0.93	7.29 ± 1.26	7.51 ± 1.25	6.31 ± 1.43	7.88 ± 1.50	7.69 ± 0.86	6.92 ± 0.88
Total ash, %	2.91 ± 0.17	3.41 ± 0.07	3.03 ± 0.12	2.97 ± 0.00	3.09 ± 0.03	3.21 ± 0.11	2.86 ± 0.24	3.10 ± 0.04

^1^ Treatments were: a control treatment without addition of dietary fiber (CON), 1% inclusion of seaweed (SEA), 1% inclusion of psyllium husk (PSY), 1% inclusion of ground chia seeds (CHI), 0.5% inclusion of seaweed and psyllium husk (SEA/PSY), 0.5% inclusion of seaweed and ground chia seeds (SEA/CHI), 0.5% inclusion of psyllium husk and ground chia seeds (PSY/CHI), and 0.33% inclusion of seaweed, psyllium husk, and ground chia seeds (SEA/PSY/CHI).

**Table 3:** pH, cooking loss, and texture profile analysis (TPA) of meat batters formulated without dietary fiber (CON) or with 1% inclusion of red seaweed (SEA), psyllium husk (PSY), and/or ground chia seeds (CHI) ^1^.

	CON	SEA	PSY	CHI	SEA/PSY	SEA/CHI	PSY/CHI	SEA/PSY/CHI	SEM	*p*-Value
pH	5.86	5.89	5.98	5.93	6.00	5.90	5.94	5.93	0.05	0.43
Cooking loss, %	0.56	0.42	0.43	0.62	0.44	0.62	0.47	0.35	0.10	0.08
Textural attributes										
Hardness, N	53.69 ^c^	71.38 ^a^	58.71 ^bc^	53.29 ^c^	66.31 ^a^	64.04 ^ab^	56.35 ^c^	56.46 ^c^	2.79	0.001
Chewiness	6.36	6.52	6.27	6.42	6.26	5.04	6.57	5.14	1.07	0.93
Cohesiveness, %	78	76	76	75	75	62	75	75	4	0.27
Adhesiveness, g·sec	−25.43	−35.18	−29.13	−26.85	−35.87	−29.58	−29.12	−35.12	4.10	0.47
Resilience, %	40	40	38	36	37	30	36	35	2	0.11

^a–c^ Least squares means within a row with different superscripts are statistically different (*p* < 0.05). ^1^ Treatments were: a control treatment without addition of dietary fiber (CON), 1% inclusion of seaweed (SEA), 1% inclusion of psyllium husk (PSY), 1% inclusion of ground chia seeds (CHI), 0.5% inclusion of seaweed and psyllium husk (SEA/PSY), 0.5% inclusion of seaweed and ground chia seeds (SEA/CHI), 0.5% inclusion of psyllium husk and ground chia seeds (PSY/CHI), and 0.33% inclusion of seaweed, psyllium husk, and ground chia seeds (SEA/PSY/CHI).

**Table 4:** Instrumental color of meat batters formulated without dietary fiber (CON) or with 1% inclusion of red seaweed (SEA), psyllium husk (PSY), and/or ground chia seeds (CHI) ^1^.

	CON	SEA	PSY	CHI	SEA/PSY	SEA/CHI	PSY/CHI	SEA/PSY/CHI	SEM	*p*-Value
Color of raw (uncooked) samples										
L*	59.67	59.08	59.15	58.54	58.79	59.75	59.00	59.05	0.76	0.77
a*	6.83	6.41	6.64	6.80	6.29	6.43	6.43	6.27	0.18	0.17
b*	16.93	15.97	16.32	16.84	16.48	16.86	16.86	15.88	0.54	0.73
ΔE*_ab_ from CON	–	2.03	0.97	1.33	1.58	0.83	1.33	1.66	0.69	0.86
Color of cooked (surface) samples										
L*	66.48 ^ab^	66.08 ^abc^	65.25 ^c^	65.53 ^bc^	66.17 ^abc^	67.02 ^a^	66.34 ^ab^	66.51 ^ab^	0.34	0.05
a*	11.42	11.90	11.78	11.52	11.79	11.35	11.68	11.42	0.33	0.89
b*	12.77	11.56	12.19	12.84	11.69	11.93	12.36	11.89	0.30	0.07
ΔE*_ab_ from CON	–	1.42	1.63	1.31	1.21	1.09	0.67	1.09	0.33	0.54
Color of cooked (internal) samples										
L*	67.37	67.37	66.83	67.40	66.72	68.05	67.38	67.68	0.32	0.13
a*	10.80	10.70	11.06	11.13	11.44	10.34	11.00	10.87	0.29	0.33
b*	11.08	10.73	10.37	11.25	10.68	10.57	10.83	10.37	0.30	0.06
ΔE*_ab_ from CON	–	2.62 ^ab^	2.47 ^ab^	1.83 ^c^	2.24 ^bc^	2.94 ^a^	2.20 ^bc^	2.75 ^a^	0.24	0.02

^a–c^ Least squares means within a row with different superscripts are statistically different (*p* < 0.05). ^1^ Treatments were: a control treatment without addition of dietary fiber (CON), 1% inclusion of seaweed (SEA), 1% inclusion of psyllium husk (PSY), 1% inclusion of ground chia seeds (CHI), 0.5% inclusion of seaweed and psyllium husk (SEA/PSY), 0.5% inclusion of seaweed and ground chia seeds (SEA/CHI), 0.5% inclusion of psyllium husk and ground chia seeds (PSY/CHI), and 0.33% inclusion of seaweed, psyllium husk, and ground chia seeds (SEA/PSY/CHI).

**Table 5:** Storage modulus (G′), loss modulus (G″), and tangent delta (δ) of meat batters formulated without dietary fiber (CON) or with 1% inclusion of red seaweed (SEA), psyllium husk (PSY), and/or ground chia seeds (CHI) ^1^.

	CON	SEA	PSY	CHI	SEA/PSY	SEA/CHI	PSY/CHI	SEA/PSY/CHI	SEM	*p*-Value
Storage modulus (G′, kPa)										
20 °C (start)	13.34	14.61	14.28	15.94	16.72	12.19	16.70	13.58	1.88	0.60
72 °C (heating)	19.69	25.04	22.80	20.72	21.19	21.06	20.61	21.54	1.41	0.28
20 °C(cooling)	51.91	53.51	40.00	50.52	58.21	42.68	49.32	41.14	7.22	0.54
Loss modulus (G″, kPa)										
20 °C (start)	9.76	11.13	12.80	13.87	9.99	10.97	13.12	8.78	2.81	0.88
72 °C (heating)	6.13	7.99	12.52	9.89	6.77	9.45	7.87	8.37	1.98	0.46
20 °C (cooling)	14.86	13.25	18.05	18.17	15.89	11.97	15.99	11.62	4.15	0.84
Tangent delta (δ)										
20 °C (start)	0.73	0.76	0.88	0.85	0.60	1.04	0.77	0.65	0.20	0.84
72 °C (heating)	0.31	0.32	0.53	0.46	0.32	0.45	0.42	0.39	0.07	0.31
20 °C (cooling)	0.28 ^bc^	0.23 ^c^	0.41 ^ab^	0.35 ^abc^	0.27 ^bc^	0.27 ^bc^	0.44 ^a^	0.28 ^bc^	0.05	0.07

^a–c^ Least squares means within a row with different superscripts are statistically different (*p* < 0.05). ^1^ Treatments were: a control treatment without addition of dietary fiber (CON), 1% inclusion of seaweed (SEA), 1% inclusion of psyllium husk (PSY), 1% inclusion of ground chia seeds (CHI), 0.5% inclusion of seaweed and psyllium husk (SEA/PSY), 0.5% inclusion of seaweed and ground chia seeds (SEA/CHI), 0.5% inclusion of psyllium husk and ground chia seeds (PSY/CHI), and 0.33% inclusion of seaweed, psyllium husk, and ground chia seeds (SEA/PSY/CHI).

**Table 6:** Molecular forces (described by the percentage of solubility of proteins using different disruptive reagents) of meat batters formulated without dietary fiber (CON) or with 1% inclusion of red seaweed (SEA), psyllium husk (PSY), and/or ground chia seeds (CHI) ^1^.

	CON	SEA	PSY	CHI	SEA/PSY	SEA/CHI	PSY/CHI	SEA/PSY/CHI	SEM	*p*-Value
Ionic razmakinteractions	3.90 ^c^	3.66 ^c^	4.01 ^c^	6.20 ^a^	5.22 ^ab^	5.49 ^a^	4.17 ^bc^	4.00 ^c^	0.42	0.003
Hydrogen razmakinteractions	29.24 ^ab^	19.47 ^b^	19.64 ^b^	31.48 ^a^	26.06 ^ab^	32.67 ^a^	32.48 ^a^	20.43 ^b^	3.44	0.04
Hydrophobic interactions	21.07	26.53	22.96	18.63	14.12	20.97	18.85	21.78	3.88	0.47
Disulfide razmakinteractions	5.31	6.46	5.12	6.20	7.69	5.99	5.82	5.40	1.08	0.60

^a–c^ Least squares means within a row with different superscripts are statistically different (*p* < 0.05). ^1^ Treatments were: a control treatment without addition of dietary fiber (CON), 1% inclusion of seaweed (SEA), 1% inclusion of psyllium husk (PSY), 1% inclusion of ground chia seeds (CHI), 0.5% inclusion of seaweed and psyllium husk (SEA/PSY), 0.5% inclusion of seaweed and ground chia seeds (SEA/CHI), 0.5% inclusion of psyllium husk and ground chia seeds (PSY/CHI), and 0.33% inclusion of seaweed, psyllium husk, and ground chia seeds (SEA/PSY/CHI).

## Data Availability

The original contributions presented in this study are included in the article. Further inquiries can be directed to the corresponding author.
